# A platform for CRISPRi-seq in *Streptomyces albidoflavus*

**DOI:** 10.1128/mbio.03065-25

**Published:** 2026-01-12

**Authors:** Justin E. Clarke, Tabitha R. Faulkner, Ryan F. Seipke

**Affiliations:** 1 Astbury Centre for Structural Molecular Biology, Faculty of Biological Sciences, University of Leeds 4468, Leeds, United Kingdom; University of Strathclyde, Glasgow, United Kingdom

**Keywords:** *Streptomyces*, CRISPRi, CRISPRi-seq, essential genes

## Abstract

*Streptomyces* produce a multitude of secondary metabolites, which have been exploited in drug discovery campaigns for more than three-quarters of a century. Our understanding of microbial physiology has been revolutionized by genome sequencing and large-scale functional studies. Technology for genome-wide investigations in *Streptomyces* species, however, has lagged behind that for other bacterial systems, hindering exploitation of unprecedented quantities of genomic data. Here, we develop a platform for *en masse* clustered regularly interspaced short palindromic repeats interference sequencing (CRISPRi-seq) for *Streptomyces* spp. By performing CRISPRi-seq with 2,160 unique sgRNAs targeting all operons (432 operons) encoding membrane transporters (629 genes) representing 1.1Mb of the 6.8Mb genome for *S. albidoflavus,* combined with hit validation, we discovered that only a small proportion (13 of 432 operons, 25 kb) contribute positively to fitness. Our work provides both a first-in-class platform for high-throughput functional genomics and a generalized blueprint for *en masse* screens in *Streptomyces* species.

**IMPORTANCE:**

*Streptomyces* bacteria are prolific producers of clinically essential natural products, yet high-throughput tools to systematically interrogate their genomes remain underdeveloped. By establishing a robust CRISPRi-seq platform for *en masse* functional screening in *Streptomyces albidoflavus*, our work closes a critical technological gap in *Streptomyces* functional genomics. Our study not only identifies a small subset of transporter operons essential for fitness but also introduces a scalable, generalizable approach for dissecting gene function. This platform will accelerate systems-level understanding of an industrially and medically important genus.

## INTRODUCTION

*Streptomyces* is one of the largest and most abundant bacterial genera in soils worldwide. They undergo a fascinating and exceptionally complex developmental cycle in which the organism progresses through a “feeding” or substrate mycelial stage, which, upon perception of poorly characterized signals, produces aerial hyphae that ultimately septate to form chains of exospores (1). Notably, a multitude of bioactive secondary or specialized metabolites are produced in response to environmental cues (2). Biotechnology has exploited many of these natural products as anticancer, antiviral, insecticidal, herbicidal, antibacterial, antifungal, and immunosuppressive compounds (3). More than two-thirds of therapeutic small molecules used in medicine are derived from or inspired by natural products produced by *Streptomyces* species and related filamentous actinobacteria (4). It is now well appreciated that the utility of these agents has been eroded over the last half-century due to misuse. Growing concerns about antibiotic resistance combined with failure to find new leads from screening of large libraries of synthetic compounds have led to a renewed interest in natural products discovery (5). This renaissance has been fueled largely by the relatively inexpensive cost to sequence genomes of strains that produce promising bioactive small molecules. For example, there are currently more than 12,500 streptomycete genomes available in GenBank compared with 10 years ago when there were only ~150 (6). Access to this genomic data has enhanced our understanding of evolution of the genus and has also led to exquisite genetic insight into important processes, such as morphological development, the perception of environmental stress, and secondary metabolism among other areas. However, overall, the field has not yet been able to truly harness the “power” of the genome sequence because there is a distinct lack of technology for high-throughput functional genomics in *Streptomyces* species, which fundamentally limits biological insights that can be drawn from genomic data alone. While transposon-based approaches are well developed in other microorganisms, it has only been recently that the problem of low transposition frequency in *Streptomyces* was overcome with the development of a hyperactive variant of Tn5 (7). Although this transposon variant is useful, it is inherently biased towards large genes and characterization of insertion sites requires the laborious recovery and sequencing of a rescue plasmid or Tn-sequencing. The latter relies upon random digestion or shearing of chromosomal DNA, which means that after bulk sequencing, the precise site of insertion may not necessarily indicate the identity of the disrupted gene.

Gene silencing by clustered regularly interspaced short palindromic repeats interference (CRISPRi) has emerged as an alternative, which employs a catalytically inactive Cas9 (dCas9) and single-guide RNA (sgRNA) to prevent expression of a target gene by blocking RNA polymerase activity. CRISPRi can be adapted for high-throughput analyses when combined with “dropout” amplicon sequencing (CRISPRi-seq), which is used to enumerate the abundance of unique sgRNAs in a mixed/competitive culture system (8, 9). CRISPRi-seq has therefore proved to be a powerful approach for functional genomics *in vitro* and *in situ;* however, its development has thus far been limited to unicellular microorganisms for which large construct libraries can be introduced by (electro)transformation relatively easily (10–13). Here, we report the development of CRISPRi-seq in *S. albidoflavus* J1074, a widely used heterologous expression strain for natural product discovery. We constructed a library of 2,160 unique sgRNAs targeting all transcriptional units (TUs) harboring putative membrane transporters. Based on this screen and individual CRISPRi validation experiments, we established that only 13 membrane transporters positively contribute to fitness in minimal medium. We anticipate our platform will serve as a powerful functional genomics tool to better understand the rules of life in *Streptomyces* species and can be exploited for rational strain breeding industrial *Streptomyces* strains.

## RESULTS AND DISCUSSION

### An integrative CRISPRi system controlled by a dual-switch for use in *Streptomyces* spp.

**Fig 1:**
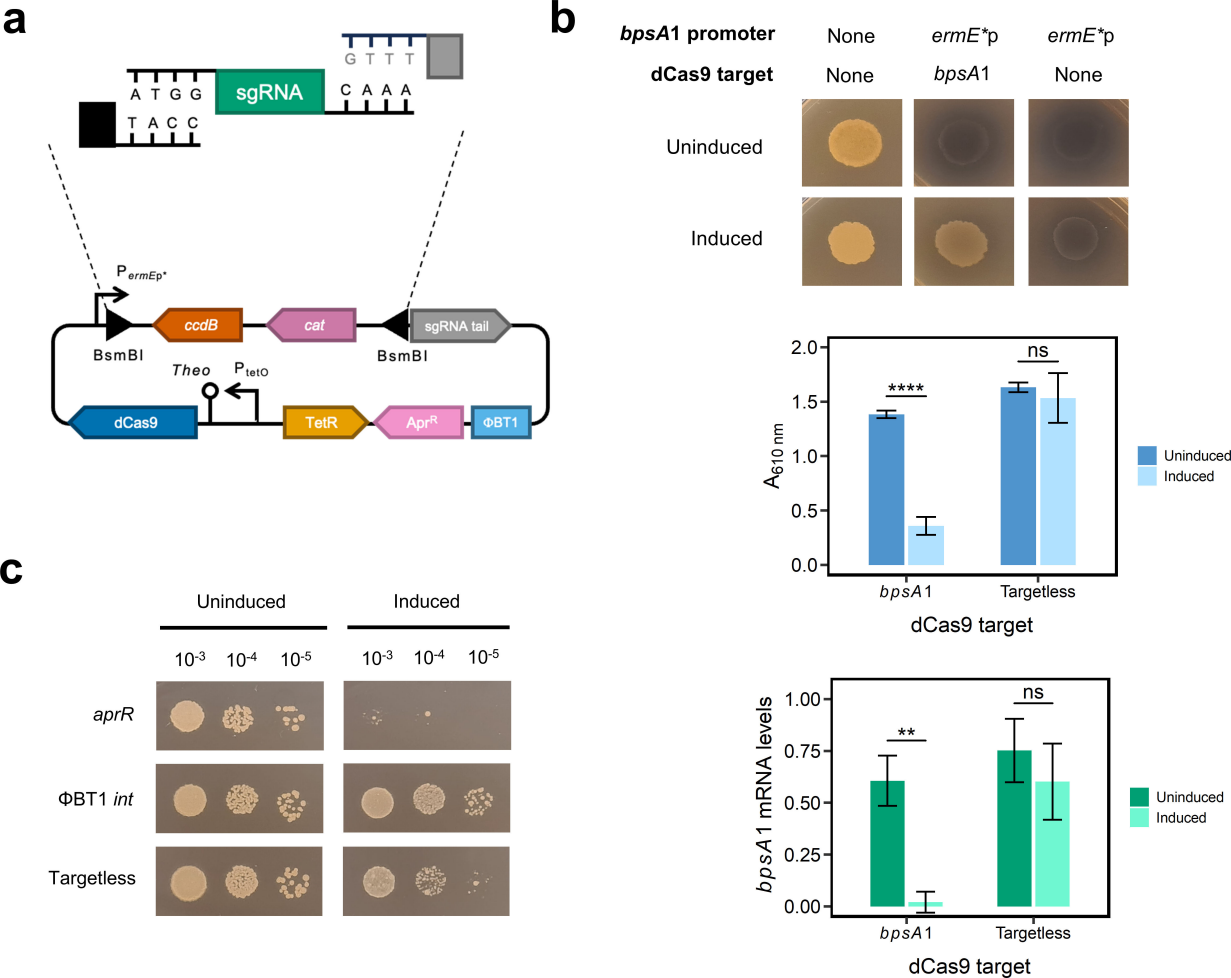
CRISPRi silencing in *Streptomyces albidoflavus*. (**a**) Diagrammatic representation of pRFSdCas9 and sgRNA cloning by Golden Gate assembly. (**b**) Silencing of the *bpsA*1 gene harbored by pIndigoC31Hyg when expressed from the medium-strength constitutive promoter ermE* using ISP-2 agar (top), and using ISP-2 broth, for which indigoidine production was quantified spectrophotometrically (middle), and *bpsA1* knockdown was verified by RT-qPCR transcript level (bottom). Error bars represent standard deviations (*n* = 3), with *P*-values calculated using a Student’s *t*-test. **** = *P* < 0.0001; ** = *P* < 0.01; ns, no significance. (**c**) Silencing of the genes for apramycin resistance (*aprR*) and ΦBT1 integrase on glucose minimal media agar with and without apramycin supplementation in comparison to uninduced controls and the targetless_4 strain.

We started by designing a new CRISPRi plasmid (pRFSdCas9) for which the key considerations included genetic stability, tight regulatory control of dCas9 expression to avoid known problems with toxicity (14, 15), and a strategy for high efficiency cloning of thousands of unique sgRNAs. pRFSdCas9 stably integrates into the ΦBT1 *attB* site of the host chromosome, which permits selection-free propagation of the library (16) and prevents cross-conjugation of plasmids once introduced. Limiting the dCas9 gene to a single copy also facilitated tighter control over its expression from a new dual switch we developed, which consisted of an anhydrotetracycline-inducible promoter and a theophylline-inducible riboswitch (aTc-theo) (17, 18). Finally, pRFSdCas9 contains two BsmBI/Esp3I restriction sites to enable seamless Golden Gate cloning of any guide RNA sequence between a constitutive promoter and the sgRNA tail sequence. The presence of a counter selection gene, *ccdB* (19), between the cloning sites enables the recovery of successful clones with minimized background (Fig. 1a). While our study was in progress, a new dCas9 plasmid with design specifications compatible with CRISPRi-seq was published (20). For our system, optimal concentrations of anhydrotetracycline (20 ng mL^-1^) and theophylline (4 mM) for induction were determined using a checkerboard assay in which the aTc-theo dual switch controlled the expression of an indigoidine synthetase reporter gene (*bpsA*) and indigoidine production was quantified spectrophotometrically (Fig. S1). The functionality of pRFSdCas9 was demonstrated by silencing expression of *bpsA* and quantifying indigoidine production and transcript abundance by RT-qPCR (Fig. 1b). The ability of CRISPRi to influence fitness in an artificial setting was tested by targeting the apramycin resistance gene of chromosomally integrated pRFSdCas9, which caused cell death in the presence of apramycin, but had no effect on viability in its absence, whereas silencing of a control gene, the ΦBT1 integrase, or use of an sgRNA with no chromosomal target did not impact viability in the presence of apramycin (Fig. 1c).

### A CRISPRi library targeting all membrane transporters

To develop CRISPRi-seq on the megabase scale, we designed a strategy to silence all TUs harboring a putative membrane transporter gene(s). Transporters were investigated because they are (i) essential for import of nutrients and export of high-value compounds during industrial fermentation, (ii) dispersed throughout a large proportion of the genome, (iii) typically encoded within their own specific TU, which would streamline data interpretation. In total, *S. albidoflavus* harbors 629 putative transporter genes organized within 432 TUs predicted from analysis of previous RNA-sequencing data (21), representing ~16% of the genome (1.1 Mbp of the 6.8 Mbp chromosome, see Tables S1 and S2).

**Fig 2:**
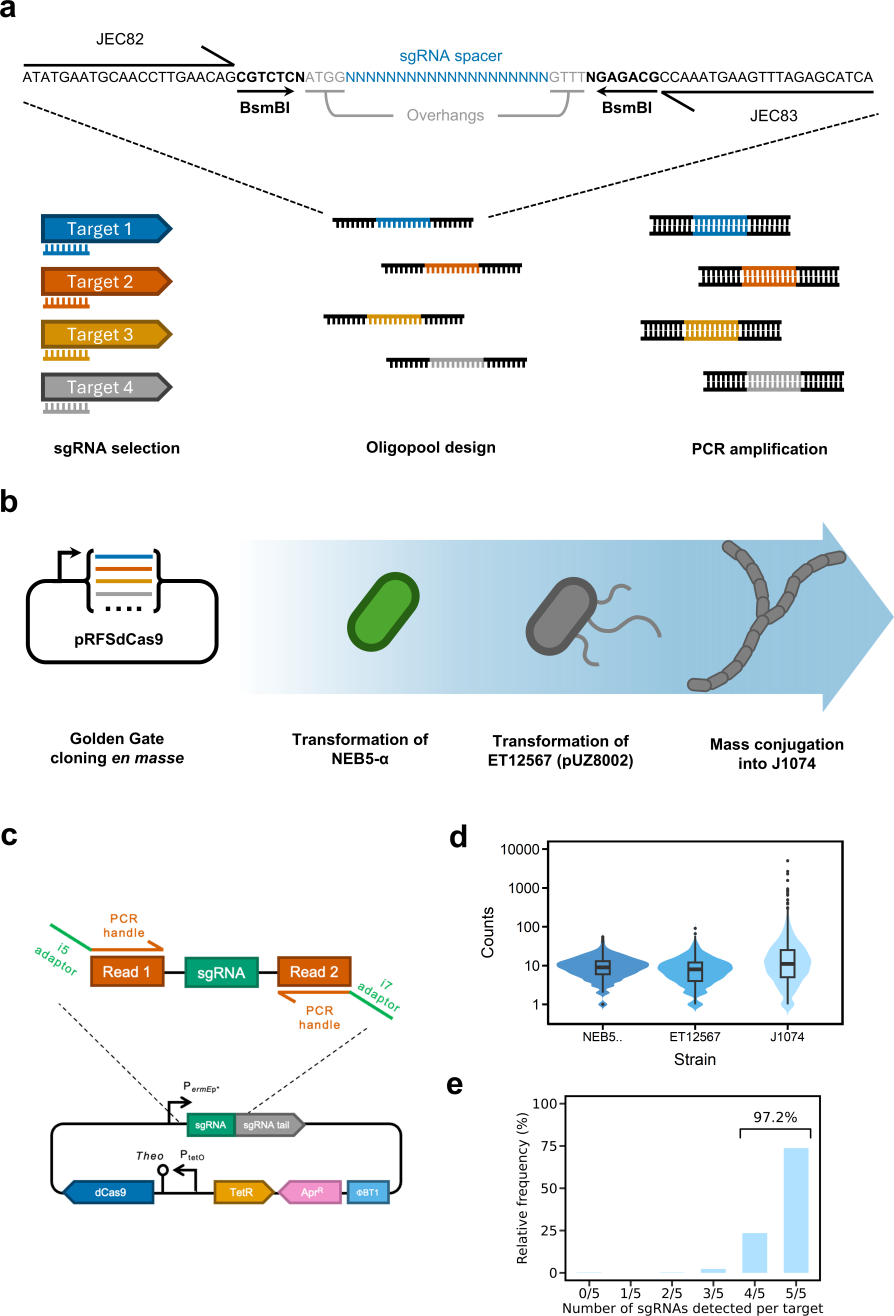
Design of an sgRNA library to target all transporter genes in *S. albidoflavus*. (**a**) Workflow for design of the oligopool. (**b**) Workflow for construction of the sgRNA library by *en masse* Golden Gate assembly and mobilization into *S. albidoflavus* J1074. (**c**) Schematic for preparation of amplicons for sequencing. (**d**) Violin plots graphically depict the counts for sgRNA clones during each stage of library construction, as determined by amplicon sequencing. (**e**), The relative number of targets that have at least four of the five sgRNAs detected within the J1074 library.

sgRNAs targeting the 5′-end (i.e.*,* first 100 bp) are known to facilitate effective CRISPRi silencing in other bacteria, including *Streptomyces* spp. (22). In line with strategy, CRISPy-web (23) was therefore used to design three sgRNAs targeting this region for each of the 432 TUs as well as two additional sgRNAs per TU that targeted anywhere within the first coding sequence, as a previous report indicated silencing in *Streptomyces* spp. may not be impacted by location of sgRNA binding (24). Thus, an oligonucleotide pool was commercially synthesized that harbored a total of 2,165 unique sgRNAs (2,160 TU-targeting sgRNAs + 5 targetless negative control sgRNAs, Table S3) with flanking sequences necessary to facilitate PCR amplification and *en masse* Golden Gate assembly into pRFSdCas9 (Fig. 2a). The resulting plasmid pool was initially established in a cloning strain of *Escherichia coli* prior to mobilization to a conjugal donor and *en masse* conjugation into *S. albidoflavus* J1074 (Fig. 2b). The heterogeneity of the sgRNA library during each stage was assessed by amplicon sequencing (Fig. 2c and d; Fig. S2). Although there was a reduction in the relative abundance for the majority of sgRNAs after mobilization into *S. albidoflavus,* an amplicon signal was detected for ~95% of all sgRNAs, likewise at least four sgRNAs were detected for 97% of targeted TUs (Fig. 2e).

### CRISPRi-seq reveals membrane transporters that contribute positively to fitness

**Fig 3:**
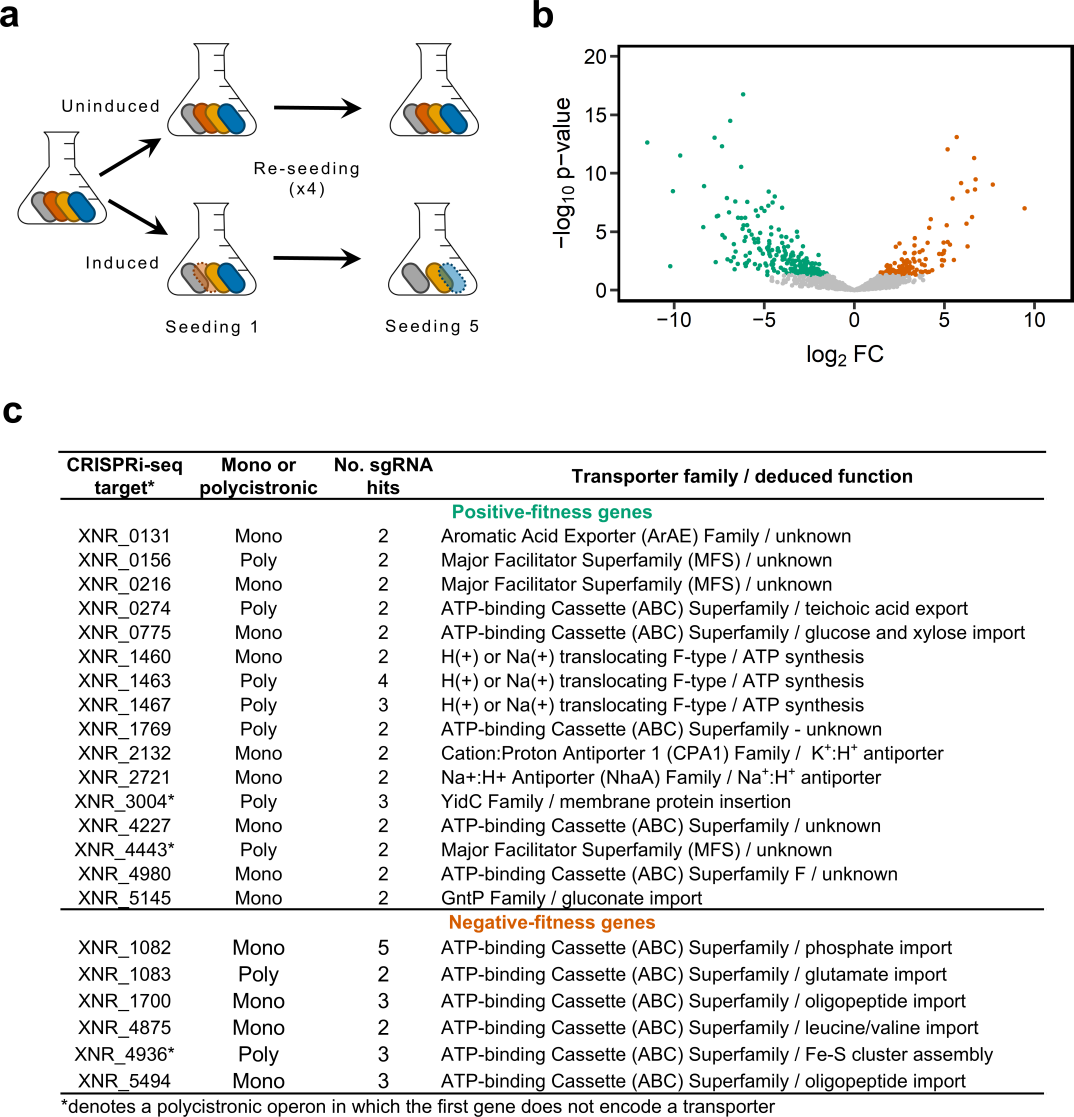
CRISPRi-seq reveals transporters that contribute to fitness. (**a**) Workflow for competition growth assays. (**b**) Volcano plot depicting the abundance for each sgRNA under induced and uninduced conditions in liquid minimal media. Green circles indicate sgRNAs that show reduced abundance; orange circles indicate sgRNAs that show increased abundance. FC, fold-change. (**c**) Transporter genes considered to contribute to fitness either positively (green) or negatively (orange), based on two or more sgRNAs per target showing a significant decrease or increase in abundance upon induction, respectively and statistically supported by an adjusted *P*-value of < 0.05.

We screened our transporter sgRNA library using minimal medium broth supplemented with glucose and asparagine in the presence and absence of anhydrotetracycline and theophylline. In accordance with other CRISPRi-seq studies (10, 11, 25, 26), after three culture re-seedings (~22 generations), sgRNA abundance was analyzed by amplicon sequencing (Table S4), which allowed examination of each TU targeted with respect to whether it contributed positively or negatively to fitness (Fig. 3). For most TUs targeted, one or fewer sgRNAs changed in abundance between induced and uninduced cultures. These TUs were thus considered fitness-neutral. For the remaining 22 TUs, 16 were classified as positive-fitness TUs, and six were classified as negative-fitness TUs on the basis that two or more sgRNAs displayed a statistically significant decrease or increase in abundance when induced versus uninduced cultures were compared, respectively (Fig. 3). Further analysis of the sgRNA hits for the positive-fitness TUs revealed that a large proportion (approximately 50%) of the binding sites were located >150 bp downstream of the putative start codon of the first gene (Fig. S3). Likewise, there was no correlation between sgRNA-binding location (with respect to start codons) and the *P*-value (Fig. S3). Taken together, these observations suggest that in J1074, CRISPRi efficiency is less dependent on sgRNA-binding location compared with other streptomycetes (22). Positive-fitness TUs are discussed in detail below.

### Validation of positive-fitness transcriptional units

**Fig 4:**
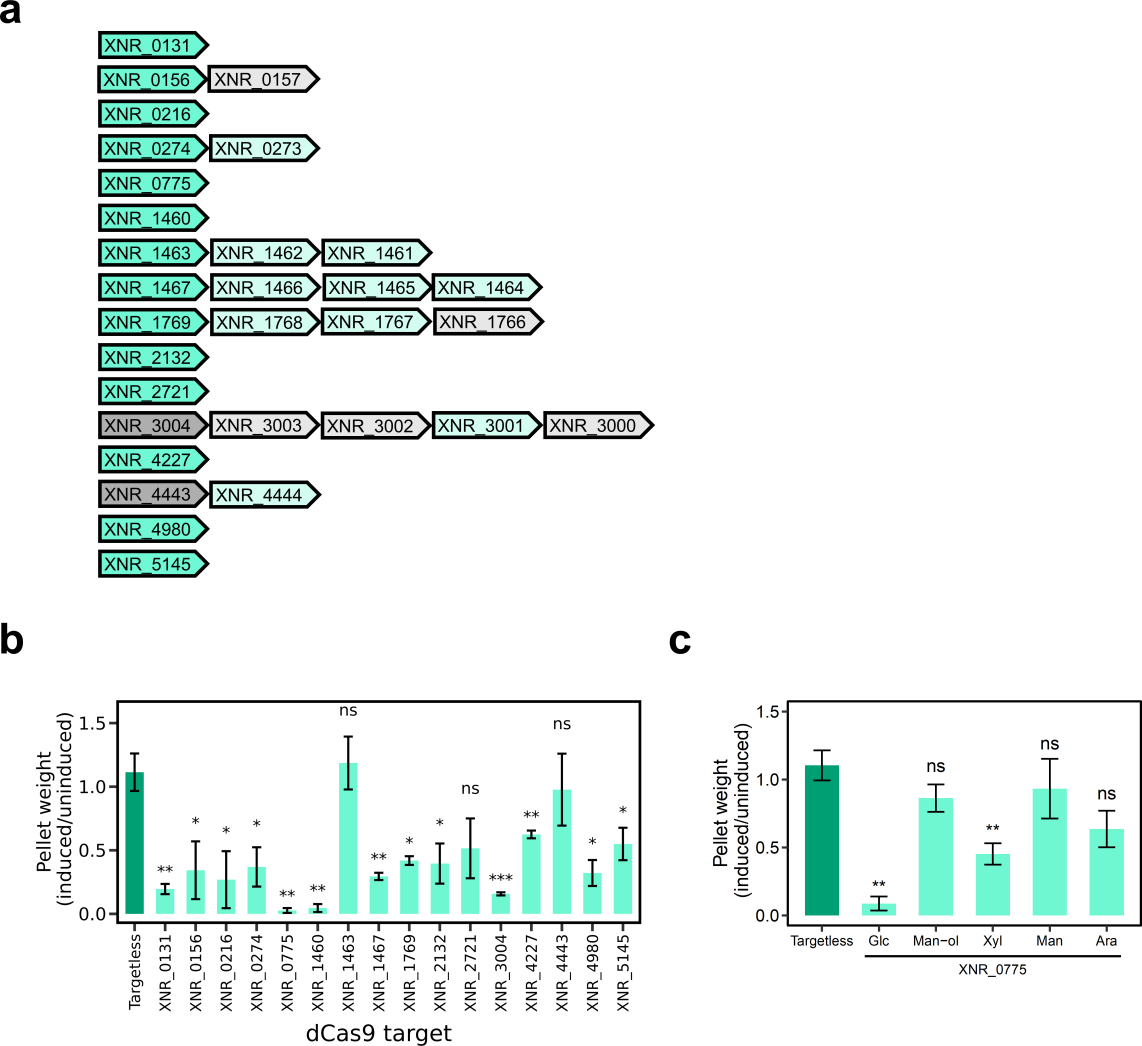
Validation of transporters that contribute positively to fitness. (**a**) Diagrammatic representation of positive-fitness transcription unit (TUs). Green arrows indicate transporter genes. Gray arrows indicate non-transporter genes. Darker color arrows indicate those targeted by CRISPRi-seq and validation experiments unless indicated by an asterisk. Annotations of all genes are displayed in Table S5. (**b**) Silencing of the 16 positive-fitness TUs in liquid minimal media. (**c**) Silencing of XNR_0775 in liquid minimal media supplemented with different sources of carbon. For both panels b and c, error bars represent the standard error of the mean (*n* = 3) with P-values calculated by a Student’s *t*-test compared with the targetless_1 control strain; *** = *P* < 0.001; ** = *P* < 0.01; * = *P* < 0.05; ns, no significance.

To establish the faithfulness of CRISPRi-seq in *S. albidoflavus*, we generated a suite of strains in which each putative positive-fitness TU was targeted by CRISPRi individually and subsequently cultivated them in monoculture using the same growth conditions employed during the screen. When CRISPRi was induced, 13 out of 16 candidates displayed a statistically significant reduction in growth compared with their respective uninduced controls, indicating that the CRISPRi-seq screen yields true hits (Fig. 3b and 4a and b).

Rationalization of several validated hits was straightforward. For instance, XNR_2132 is a CPA1-family K^+^:H^+^ antiporter, and its ortholog in *S. coelicolor* is essential for K^+^ stress and maintenance of cytoplasmic pH (27, 28). Also, the TU comprising XNR_0273 and XNR_0274 encodes orthologs of TagH and TagG, respectively, for translocation of teichoic acids produced by adjacent genes XNR_0275-to-XNR_0279, which are components of the cell wall that are important for its function and integrity (29). Interestingly, glucose was the sole carbohydrate used in our CRISPRi-seq screen, which identified two putative carbohydrate transporters (XNR_5145 and XNR_0775) orthologous to characterized *S. coelicolor* transporters for oxidized glucose (i.e., gluconate) and xylose, respectively (30, 31). While we expected the former to be a hit, we were surprised that XNR_0775 (a putative xylose substrate binding protein) was identified given the absence of xylose in our screen. Targeting XNR_0775 with CRISPRi did not impact growth when glucose was substituted with alternative carbon sources, including mannitol, mannose, and arabinose, while reduced growth was observed with glucose and when it was substituted with xylose (Fig. 4C). Taken together, these data indicate that XNR_0775 is a substrate binding protein whose substrates include glucose and xylose.

Three TUs, XNR_1460 (ε-subunit), XNR_1463-to-XNR_1461 (⍺-, β-, γ-subunits), XNR_1467-to-XNR_1464, (a-, b-, c-, δ-subunits) encode components of the F_0_F_1_-ATP synthase (Table S5; Fig. S4), which is the primary driver of ATP production conserved across all domains of life and orthologous genes were identified by Tn-sequencing as essential in *S. coelicolor* (7). CRISPRi targeting of XNR_1460 or XNR_1467-to-XNR_1464 compromised growth in minimal medium broth (Fig. 4B); however, to our surprise, targeting of XNR_1463-to-XNR_1461 only compromised growth in minimal medium agar (Fig. S5). These data indicate that the proton pumping and maintenance of membrane potential functions performed by the F_0_ complex are critical for growth in both mono- and mixed-culture regardless of the media type. These data also suggest that the F_1_ component ATP synthase subunit may be dispensable for monoculture in minimal medium broth, presumably because substrate-level phosphorylation is sufficient to meet energy demands, whereas growth atop agar is accompanied by gradients of oxygen, nutrients, and waste products, and an overall more complex metabolic demand such that reduced capacity for oxidative phosphorylation is deleterious. It therefore follows that in mixed culture, the ability to perform oxidative phosphorylation would provide a competitive advantage.

CRISPRi targeting and measurement of biomass was insufficient to validate two CRISPRi-seq hits (XNR_4443 to XNR_4444 and XNR_2721). XNR_4443 to XNR_4444 is a bicistronic operon adjacent to the recently discovered mansouramycin D biosynthetic gene cluster (32, 33). XNR_4444 encodes an MFS1 family transporter (Table S1 and S5), and deletion of XNR_4443 reduced mansouramycin D production by ~50% (33). XNR_4443 belongs to the ATP-dependent amino acid ligase protein family and contains an ATP-grasp domain (InterProScan accessions IPR052032, IPR011761, respectively), a signature of enzymes well known to perform ligation of small molecules (34). It is thus plausible that XNR_4443 may perform the proposed conjugation reaction between 2-amino-1-(1H-indol-3-yl)ethenone and 5-(methylamino)−4-oxo-1H-pyridine-3-carboxamide during mansouramycin D biosynthesis (33, 35) and that when expression of XNR_4443 is silenced during mixed culture, accumulation of one or both intermediates may be deleterious to the producer. XNR_2721 encodes a putative NhaA family Na^+^:H^+^ antiporter whose ortholog in *S. coelicolor* (SCO3564) is involved in response to alkaline stress (27). Culture pH was not measured prior to CRISPRi-seq; however, we retrospectively confirmed with fresh cultures that neutral pH was maintained in buffered MM broth for both the whole sgRNA library and the XNR_2721 strain. While it was not statistically significant, accumulation of biomass on average reduced when silencing of XNR_2721 was induced compared to uninduced, suggesting this is likely a complicated phenotype to observe using a biomass accumulation method. While we cannot exclude the possibility that the reason the XNR_4443-to-4444 and XNR_2721 sgRNAs dropped out was because they produced off-target effects that were deleterious in mixed-culture, the fact that at least two sgRNAs for these targets were identified would suggest that this is unlikely, and in fact, these two examples exemplify the power of CRISPRi-seq, in that it not only identifies pronounced phenotypes, but it can also identify hits with phenotypes that would be otherwise missed using traditional screens, such as those using arrayed libraries with pure cultures.

### Conclusion and perspectives

CRISPRi-seq has emerged from the combination of CRISPRi and next-generation sequencing, resulting in the ability to tune gene expression on a global scale. The use of CRISPRi-seq has thus far been limited to unicellular bacteria because of the requirement for facile transformation methods for introduction of multitudinal sgRNA libraries (10–13), and hence, there is a distinct lack of CRISPRi-based genomic studies of bacteria with multicellular forms, complex developmental cycles, and/or poor genetic tractability. Here, for the first time, we established CRISPRi-seq in *Streptomyces* spp., a genus of multinuclear, filamentous, spore-forming bacteria that are a major source of high-value compounds. By targeting a subset of genes in *S. albidoflavus* J1074 involved in membrane transport, we were able to validate the feasibility of our approach. Remarkably, only 16 of the 432 TUs encoding membrane transporters contributed positively to fitness under our screen conditions.

When interpreting data from CRISPRi-seq screens, it is important to be aware of potential limitations. For instance, it is impossible to know whether the level of knockdown is comparable between targets simply because it is impractical to experimentally assess knockdown efficiency or off-target effects for thousands of sgRNAs. However, one’s bet can be hedged by using design algorithms to identify multiple high-scoring sgRNAs per target. However, in the end, statistically significant hits should conceptually be treated the same rather than drawing hierarchical conclusions without further experimentation. In addition, it is imperative that thought be given to what constitutes a CRISPRi-seq hit and what it means. Here, we were relatively conservative and considered hits only if two or more sgRNAs showed a change in abundance between treatments supported by an adjusted *P*-value of less than 0.05; our validation experiments suggest this was a sensible starting point; however, it is likely that a screen of a different nature may require a different threshold. In addition, it is important to remember that CRISPRi-seq occurs in competitive co-culture over ~20 generations, which means that the phenotypes reported fall on a spectrum ranging from profound, to subtle, to screen-specific because of microenvironmental conditions. In our study, most hits were validated by CRISPRi silencing in monoculture; however, silencing of three of our hits presumably resulted in a subtle decrease in competitiveness detectable only in competitive cultures, but we cannot eliminate the possibility that sgRNA off-target effects may have contributed.

We expect that adoption of CRISPRi-seq methods will enable genome-level investigations into *Streptomyces* spp. at timescales previously impossible; to aid its future development in filamentous Actinobacteria, we produced a step-by-step protocol for construction of sgRNA libraries and their mass mobilization into *Streptomyces* species (File S1). We anticipate that the transporter CRISPRi-seq library developed here will emerge as a powerful tool for rational breeding industrial production strains of high-value chemicals because it can be used to optimize growth on inexpensive feedstocks and to identify transporters involved in the efflux of inhibitory toxins and/or products to increase chemical tolerance and maximize production titer.

## MATERIALS AND METHODS

### Growth media, strains, plasmids, and other reagents

*Escherichia coli* strains were cultivated using Lennox agar (LA) or broth (LB). *Streptomyces* strains were propagated using either mannitol soya-flour agar (SFM, International Streptomyces Project Medium-2 agar or broth, or minimal media supplemented with 10 g L^-1^ glucose and 0.5 g L^-1^ asparagine as described (36). Culture media were supplemented with antibiotics as required at the following concentrations: apramycin (50 μg mL^-1^), carbenicillin (50 μg mL^-1^), hygromycin (50 μg mL^-1^), kanamycin (25 μg mL^-1^), nalidixic acid (25 μg mL^-1^). Oligonucleotides were purchased from Integrated DNA Technologies, and enzymes were purchased from New England Biolabs unless otherwise stated. The DNA constructs, bacterial strains, and oligonucleotides used in this study are listed in Tables S6 and S7, respectively.

### Construction of the indigo reporter plasmid

The indigoidine synthetase gene (*bpsA1*) from *S. lavenduale* was codon optimized for *S. coelicolor,* synthesized, and cloned into pTwistAmp by Twist Biosciences to yield pTwistAmp-bpsA1. In addition to the *bpsA1* gene, the synthetic construct included a KpnI site just upstream of the ATG start codon for linearization of the construct for cloning in a counterselection cassette (see below), as well as a BamHI, EcoRI, HindIII, XbaI multi-cloning site immediately downstream of the *bpsA1* stop codon. Next, the ΦC31 phage integrase gene and its cognate *attP* site were synthesized by Genscript to lack BamHI, EcoRI, HindIII, XbaI, BsaI, and BsmBI restriction sites and cloned into the EcoRI-HindIII sites of pUC57 to yield pUC57-C31. The ΦC31-*attP* cassette was released from pUC57-C31 by EcoRI-HindIII restriction, gel purified, and cloned with T4 DNA ligase into pTwistAmp-bpsA1 restricted by the same enzymes to result in pTwistAmp-bpsA1-C31. Next, a hygromycin resistance gene cassette harboring a conjugal origin of transfer was synthesized as a gBlock by Integrated DNA Technologies (IDT); the cassette was codon optimized for *B. subtilis* (note: *Streptomyces* codon optimization was not possible), and TTA codons and the restriction site for BsaI were disallowed. The Hyg-oriT cassette was used as a PCR template with RFS829/830 for disruption of the *bla* gene of pTwistAmp-bpsA1-C31 using RecET recombineering with *E. coli* GB05-red (36). The resulting recombinant plasmid was multimeric and was linearized with KpnI and treated with T4 DNA ligase under dilution conditions to promote self-ligation to yield monomeric pTwistAmp-bpsA1-C31-Hyg. Finally, the *cat-ccdB* counterselection cassette from pDGE76 (AddGene plasmid #80578)(37) was re-synthesized as a gBlock by IDT to omit EcoRI, BamHI, and BsmBI sites. The *cat-ccdB* cassette was PCR amplified using RFS831/832, and the resulting 1.5 kb fragment was gel purified and cloned into KpnI-linearized pTwistAmp-bpsA1-C31-Hyg via NEBuilder HiFi DNA Assembly. The resulting assembly reaction was used to transform *E. coli* OneShot *ccdB* Survival 2T1^R^ (Invitrogen). The final clone was named pIndigoC31Hyg (sequence available from: https://doi.org/10.6084/m9.figshare.27079864.v1).

### Construction of CRISPRi plasmid

The design of pRFSdCas9 was based on pCRISPR-dCas9 from (23) and was constructed over several steps. First, a synthetic DNA fragment harboring several features was synthesized and cloned into the EcoRI-HindIII sites of pUC57 by GenScript to yield pUC57-BT1. The features of the synthetic DNA fragment were as follows: EcoRI-[ermE*P (38)]-[AeBlue chromoprotein (iGEM part BBa_K864401) flanked by BsmBI/Esp3I sites]-[CRISPR-Cas9 array]-ΦBT1 *attP* and integrase]-HindIII. Second, an IDT gBlock was synthesized, which contained the TetR repressor and anhydrotetracycline inducible promoter, *tcp830* from reference 17 fused to a theophylline riboswitch A (18). This gBlock served as a PCR template for RFS844/845. The resulting PCR product was assembled with dCas9 from an NdeI-EcoRI fragment from pCRISPR-dCas9 (23) and EcoRI-linearized pUC57-BT1 via NEB HiFi DNA Assembly to yield pUC57-BT1-TetTheo-dCas9. Third, an Apr-oriT cassette based on pIJ773 (39) was synthesized but designed to lack Flp recombinase FRT sites. This cassette was amplified by PCR using RFS829/830. The Apr-oriT cassette was used as a PCR template with RFS829/830 for disruption of the *bla* gene of pUC57-BT1-TetTheo-dCas9 using RecET recombineering with *E. coli* GB05-red (40). The resulting recombinant plasmid was multimeric and was linearized with XbaI and treated with T4 DNA ligase under dilution conditions to promote self-ligation to yield pUC57-BT1-TetTheo-dCas9-Apr. Finally, the Aeblue chromoprotein preceding the CRISPR array was replaced with the *cat-ccdB* counterselection cassette described above using RecET recombineering. Briefly, OneShot *ccdB* Survival 2T1^R^ was transformed with pSC101-P_BAD_-ETgA-tet and prepared for recombineering as described (40), and subsequently co-transformed with pUC57-BT1-TetTheo-dCas9-AproriT and a *cat-ccdB* cassette PCR amplified with RFS846/847. Finally, two native BsmBI sites discovered within the final construct were removed as follows. JEC94 and JEC95, containing an internal EcoRI site, were hybridized and introduced into pRFSdCas9 by Golden Gate cloning using BsmBI/Esp3I and T4 DNA ligase. The reaction consisted of just one cycle of digestion at 37°C for 10 min and ligation at 16°C for 10 min, and the final reaction mixture was used to transform *E. coli* OneShot *ccdB* Survival 2T1^R^ (Invitrogen). The resulting plasmid, pRFSdCas9EcoRI, was linearized with EcoRI. JEC96 was cloned into EcoRI-linearized vector via NEBuilder HiFi DNA Assembly, resulting in removal of the additional BsmBI/Esp3I sites. The final plasmid was named pRFSdCas9 (Fig. 1A; sequence available from: https://doi.org/10.6084/m9.figshare.27079864.v1).

### Titration of anhydrotetracycline and theophylline for dCas9 induction

For pIndigoC31Hyg-aTc-Theo, a DNA fragment consisting of TetR-*theo* flanked by BsaI sites was amplified by PCR using pRFSdCas9 as a template and oligonucleotides JEC41 and JEC42. The resulting PCR product was introduced into pIndigoC31Hyg by Golden Gate cloning using BsaI and T4 DNA ligase. The final reaction product was used to transform *E. coli* NEB 5-⍺. Successful clones were introduced into ET12657/pUZ8002, followed by conjugation into *S. albidoflavus* J1074 to generate the final strain J1074 aTc-Theo-Indigo. For pIndigo-ermE*p, a DNA fragment consisting of ermE*P flanked by BsaI sites was synthesized by IDT and introduced into pIndigoC31Hyg by Golden Gate cloning using BsaI and T4 DNA ligase. Successful clones were conjugated into J1074 to generate the final strain J1074 ermE*p-Indigo. For sgRNA constructs, single spacer inserts were generated by annealing two oligonucleotides as shown in Table S1. The forward oligonucleotide consisted of 4 nt (5′-ATGG) followed by the guide RNA sequence. The reverse oligonucleotide consisted of 4 nt (5′-AAAC) followed by the reverse complementary sequence to the guide RNA. The final annealed product was introduced into pRFSdCas9 by Golden Gate cloning using Esp3I and T4 DNA ligase. Successful clones were conjugated into J1074 ermE*p-Indigo. For solid media, spore stocks were diluted to approximately 10^8^ spores mL^−1^ in PBS. Then, 10 uL of each diluted stock was spotted onto ISP-2 agar supplemented with 50 mM HEPES pH 7.5, 50 ng mL^-1^ anhydrotetracycline, and 4 mM theophylline. Plates were allowed to dry in a sterile cabinet and were then incubated at 30°C for 48 h. For liquid assays, approximately 10^6^ spores were inoculated into 50 mL ISP-2 containing 50 mM HEPES pH 7.5. Inducers were added to a final concentration of 20 ng mL^-1^ anhydrotetracycline and 4 mM theophylline. Cultures were incubated at 30°C, 200 rpm for 48 h. Mycelia were harvested by centrifugation of 2 mL of culture at 4,000 × *g* for 10 min. The absorbance at 610 nm of each supernatant was measured in a Fluorstar plate reader and was normalized by wet mycelial weight.

### Total RNA extraction and reverse transcription quantitative PCR

For liquid assays, mycelial pellets were resuspended in 400 µL of 50 mM Tris-Cl pH 8, followed by addition of lysozyme to a final concentration of 10 mg mL^-1^. For samples grown on solid media, patches of growth were transferred from the agar to 400 µL of 50 mM Tris-Cl pH 8 using a sterile pipette tip. Patches were suspended in the solution by thorough vortexing before addition of lysozyme. Reactions were incubated at room temperature for 10 min, followed by addition of 400 µL RNA Lysis buffer provided in the Monarch Total RNA Miniprep kit (New England Biolabs). Samples were vortexed for 10 s, and then lysate was cleared by centrifugation at 16,000 × *g* for 2 min. Lysate was transferred to a gDNA Removal column and purification of total RNA was performed using the Total RNA Miniprep kit as per the manufacturer’s instructions. A further in-tube DNase I digestion was performed on the final eluate using TURBO DNase (ThermoFisher) as per the manufacturer’s instruction. Total RNA was then repurified using the Total RNA Miniprep kit. The integrity of the RNA was confirmed by separation of 500 ng of each sample on an agarose gel. RT-qPCRs were performed using the GoTaq one-step RT PCR kit (Promega) with 100 ng of total RNA and oligonucleotides (Table S7) as per the manufacturer’s instructions. All RNA samples included a negative control reaction without reverse transcriptase. A calibration standard was also performed using a 10-fold dilution series (ranging from 1 ng to 1 fg) of a cDNA template, which was PCR amplified using the same primers and genomic DNA isolated from J1074. All RNA samples also included an internal control reaction using JEC90/91 primers specific to the *rpoB* transcript, which also included a separate calibration series. Reactions were performed on a CFX RT-PCR machine with the following parameters: samples were incubated at 50°C for 10 min, then 35 cycles of 95°C for 30 s followed by 60°C for 30 s, and a final step consisting of a slow ramp to 95°C to obtain thermal melt curves. Cq values for each sample were converted to transcript levels (ng) using the calibration series, and then background subtractions were performed using the negative RT values. Finally, transcript levels were normalized using the *rpoB* transcript levels to obtain final expression levels.

### Design and construction of the sgRNA library

Rockhopper v2.0.3 (41) was used to predict composition of transcriptional units from existing RNA-sequencing data (E-MTAB-7715 [20]). The sgRNAs targeting transcriptional units harboring TransportDB-identified transporters (Tables S1 and S2) were designed using CRISPy-web using the default settings (22). The five best-scoring sgRNAs targeting non-template strand were manually selected and curated (2,160 sgRNAs); five targetless sgRNAs were also designed (Table S3). One targetless sgRNA targeted the codon-optimized *bspA* gene (above), and the remaining four targetless sgRNAs were designed by generating a random 20 bp sequence using the Random DNA Sequence Generator (http://www.faculty.ucr.edu/~mmaduro/random.htm) using a GC content of 50%. BlastN analysis was used to ensure that nucleotides comprising the 12 bp sgRNA seed sequence (i.e.*,* nucleotides 8–20 of the sequence) did not possess homology with the *S. albidoflavus* J1074 genome. The total library of 2,165 sgRNA sequences was synthesized as an OligoPool (GenScript) each flanked by BsmBI/Esp3I sites and footholds for PCR amplification. The pool was PCR amplified using JEC82/83 and introduced into pRFSdCas9 by Golden Gate cloning using BsmBI/Esp3I and T4 DNA ligase. A total of 16 reactions were performed to ensure sufficient levels of each clone were obtained. The reactions were pooled and used to transform *E. coli* NEB 5-⍺, resulting in a total of 60 transformations and a total number of approximately 138,000 colonies (approximately 64 colonies per clone). Colonies from each plate were resuspended in 2 mL LB using a sterile spreader. Suspensions were pooled and aliquoted into cryovials and stored as glycerol stocks at −80°C. Approximately 1.1 × 10^9^ CFUs from the glycerol stocks were inoculated into 20 mL of LB broth supplemented with 50 µg mL^-1^ apramycin, which was incubated at 37°C, with shaking at 200 rpm for 14–16 h. Plasmid libraries were obtained using Monarch Plasmid Miniprep kits (New England Biolabs) and used to transform ET12567/pUZ8002 (42), resulting in a total of 40 transformations and a total number of approximately 212,000 colonies. Libraries were prepared and stored as mentioned previously. Approximately 5.5 × 10^10^ CFUs from the glycerol stocks were inoculated into 1 L LB broth supplemented with 50 µg mL^-1^ apramycin, 25 µg mL^-1^ chloramphenicol, and 25 µg mL^-1^ kanamycin, which was incubated at 37 °C, with shaking at 200 rpm for approximately 12 h until the OD_600 nm_ reached 0.6. Cells were harvested by centrifugation at 4,000 × *g* for 10 min, washed three times in 300 mL LB broth, and resuspended in 30 mL LB broth. Approximately 5 × 10^10^ J1074 spores were added to the suspension, and the contents were harvested by centrifugation. Pellets were resuspended in 10 mL LB broth and plated in 300 µL aliquots on SFM agar. Plates were incubated at 30°C for 17 h. Plates were then flooded with apramycin and nalidixic acid and incubated at 30°C for 48 h. Transconjugants from each plate were resuspended in 3 mL 20% (v/v) glycerol using a sterile swab. Suspensions were pooled and aliquoted into cryovials and stored at −80 °C.

### Tracking sgRNA library construction and mobilization

Cultures of the J1074 library were prepared by inoculation of 1.1 × 10^9^ spores into 50 mL ISP-2 broth supplemented with 50 µg mL^-1^ apramycin and 25 µg mL^-1^ nalidixic acid and incubated at 30°C, with shaking at 200 rpm for 48 h. Mycelia were harvested by centrifugation of 5 mL of culture at 4,000 × *g* for 10 min, and pellets were resuspended in 200 µL P1 buffer (Qiagen). Lysozyme was added to a final concentration of 10 mg mL^-1^, and reactions were incubated at 37 °C for 2 h until lysis was observed. NaCl and SDS were added at final concentrations of 1.25 M and 1% (v/v), respectively. Samples were inverted several times and incubated on ice for 20 min. Samples were subjected to phenol extraction using Tris-saturated phenol:chloroform:isoamyl alcohol (25:24:1) until the aqueous phase was clear. DNA was precipitated by the addition of 2.5× volumes of absolute ethanol to each aqueous phase and was subsequently pelleted by centrifugation at 16,000 × *g* for 10 min. Pellets were washed twice with 70% (v/v) ethanol and allowed to air dry in a fume cupboard for 30 min, before being resuspended in sterile water. To enrich for DNA containing the protospacer region, 100–200 µg of genomic DNA was digested with 100 U SbfI (New England Biolabs) at 37°C for 12 h, followed by separation on agarose gels and excision of DNA fragments between 3,000 and 5,000 bp in length. Final gel-purified fragments were used as DNA template for amplicon-seq. For NEB 5-⍺ and ET12567/pUZ8002 clone libraries, plasmids were purified as mentioned previously and used as the DNA template. The protospacer region was PCR amplified using JEC134/135 and 500 ng DNA template. PCR products were purified via gel electrophoresis and sequenced using the Amplicon-EZ sequencing service (~100,000 reads per sample) (Azenta Life Sciences). Data were processed using the 2FAST2Q program developed previously for CRISPRi sequence read counts (9, 43). Analysis was performed using “Counter” mode on reverse reads, due to the short distance between the Illumina adaptor and the protospacer, which resulted in higher quality sequencing on this strand (8). Using ggplot2, counts per clone were presented as violin plots for each of the three libraries (44).

### Dropout of sgRNAs during cultivation in minimal medium

Starter cultures in ISP-2 were prepared as described previously. Mycelia were harvested from 1 mL of culture by centrifugation and washed in 1 mL minimal media supplemented with glucose (MM-Glu) twice. Mycelia were resuspended in 1 mL MM-Glu and inoculated into 50 mL MM-Glu supplemented with 50 mM HEPES pH 7.5. Similar cultures were prepared supplemented with 20 ng mL^-1^ anhydrotetracycline and 4 mM theophylline. Cultures were incubated at 30°C, with shaking at 200 rpm for 3–5 days. Libraries were sub-cultured by the addition of 1 mL of culture into fresh MM-Glu, followed by further incubation. The sub-culturing step was repeated twice, resulting in a final number of generations of approximately 22. Genomic DNA was isolated, and the sequence containing the protospacer region was prepared as mentioned previously, except that a second nested PCR was performed on the initial amplicon using JEC196/197 to reduce the final product length. Sequencing libraries were prepared by two-step PCR of the amplicons to introduce 3′ adaptor sequences, followed by sequencing on a NextSeq2000 v3 lane using P2 reagents (approximately 24 million reads per sample). Data were processed and analyzed as mentioned previously. Fitness of each clone upon induction was assessed using the DESeq2 package in R, as described in previous work (https://github.com/veeninglab/CRISPRi-seq) (8, 9). Positive- or negative-fitness targets were defined as those whereby at least two sgRNA clones showed significant difference in counts upon induction with an adjusted *P*-value (Padj) of less than 0.05.

### Validation of CRISPRi-seq hits

To validate positive fitness targets, individual CRISPRi constructs were made by Golden Gate assembly and introduced into *S. albidoflavus* J1074 as described above. Approximately 10^8^ spores were used to inoculate liquid minimal media supplemented with HEPES pH 7.5 (50 mM) in the presence and absence of inducers. Cultures were incubated at 30 °C, 180 rpm for 2–3 days, and growth was measured as wet mycelial mass obtained from 2 mL of culture. To test silencing of XNR_1463-to-XNR_1461 while grown on agar, spore stocks were thawed and diluted in sterile PBS as 10-fold dilution series and spotted onto MM agar supplemented with HEPES pH 7.5 (50 mM), nalidixic acid (25 µg mL^-1^) and apramycin (50 µg mL^-1^) in the presence and absence of inducers. Plates were incubated at 30°C for 5–6 days.

## Data Availability

Data generated during this study and its analysis are included within the article and supplemental material. All raw sequence data have been deposited in the NCBI Short Read Archive under accession PRJNA1159755. The *S. albidoflavus* J1074 genome sequence is publicly available in Genbank under accession CP004370.1. pRFSdCas9 and pIndigoC31Hyg sequences are available via FigShare at https://doi.org/10.6084/m9.figshare.27079864.v1. Other data are available from the corresponding author upon reasonable request.

## SUPPLEMENTAL MATERIAL

File S1 mbio.03065-25-s0001.docx Protocol for constructing CRISPRi-seq libraries in *Streptomyces* spp.

Supplemental Figures mbio.03065-25-s0002.docx Figures S1–S5 and captions for supplemental tables.

Supplementary Tables mbio.03065-25-s0003.xlsx Tables S1–S7.

ASM does not own the copyrights to Supplemental Material that may be linked to, or accessed through, an article. The authors have granted ASM a non-exclusive, world-wide license to publish the Supplemental Material files. Please contact the corresponding author directly for reuse.
