# Supplementary material for: A platform for CRISPRi-seq in Streptomyces albidoflavus
Source: mBio. 2026 Jan 12;17(2):e03065-25. doi: 10.1128/mbio.03065-25 (PMC12892944; doi:10.1128/mbio.03065-25)
Supplement: File S1 — Protocol for constructing CRISPRi-seq libraries in Streptomyces spp. [file mbio.03065-25-s0001.docx]

**Protocol for *en masse* CRISPRi library generation in *Streptomyces spp.***

**Oligopool design**

To design sgRNA sequences, we use CRISPy-web (<https://crispy.secondarymetabolites.org/#/input>) for less than 500 targets. We recommend the following criteria when designing CRISPRi libraries:

- The targets should be the first gene in a transcriptional unit (TU), based on mapped transcriptomic data.
- At least five sgRNAs should be designed per target.
- All sgRNAs sequences should target the non-template strand.
- At least three of these sgRNAs should be designed to bind within the first 150 bp of the target CDS.
- Try to avoid sgRNA sequences that have off targets with one or less mismatch.

To each sequence, place the following flanking sequences:

5’ end ATATGAATGCAACCTTGAACAG**CGTCTCN**ATGG

3’ end GTTT**NGAGACG**CCAAATGAAGTTTAGAGCATCA

Oligopools can be synthesised using a commercial service.

1. Setup PCR reactions as follows:

| Component | Stock concentration | Final amount | Volume |
| --- | --- | --- | --- |
| Q5 reaction buffer  dNTPs  JEC82  JEC83  Oligopool  Q5 DNA Polymerase  Water | 5x  10 mM each  10 µM  10 µM  10 ng/µL  2 U/µL  - | 1x  0.5 mM each  0.5 µM  0.5 µM  10 ng  2 U  - | 20 µL  2 µL  5 µL  5 µL  1 µL  1 µL  To 100 µL |

1. Perform following reaction in thermal cycler:

| 98°C for 30 s |  |
| --- | --- |
| 98°C for 30 s |  |
| 60°C for 30 s | Repeat 34x |
| 72°C for 30s |  |
| 72°C for 2 min |  |
| 4°C hold |  |

1. Separate reactions via agarose gel electrophoresis.
2. Perform gel extraction using commercial kit.
3. Quantify DNA fragment via UV spectrophotometry.

**Golden Gate Assembly**

To ensure high coverage of the sgRNA library, we typically aim for >50 colonies per sgRNA clone. We generally find we obtain 2000-3000 colonies per Golden-Gate reaction, but it is recommended that a single Golden-Gate reaction and transformation is performed first to test the efficiency of both steps.

1. Setup Golden Gate assembly reactions as follows:

| Component | Stock concentration | Final amount | Volume |
| --- | --- | --- | --- |
| PCR product  pRFSdCas9  Esp3I  T4 DNA ligase  T4 DNA ligase buffer  Water | -  -  10 U/μL  400 U/μL  10x  - | 17 ng  50 ng  5 U  200 U  1x  - | -  -  0.5 μL  0.5 μL  1 µL  To 10 μL |

1. Perform following reaction in thermal cycle:

| 37°C for 10 min | Repeat 29x |
| --- | --- |
| 16°C for 10 min |  |
| 37°C for 10 min |  |
| 65°C for 20 min |  |

Reactions can be stored at -20°C before transformation of chemically competent *E. coli*

**Chemically competent *E. coli* preparation**

We generally make our chemically competent *E. coli* strains fresh to ease the process of aliquoting and pooling of samples during library preparation. One culture of 50 mL will give 25x 100 µL aliquots,

1. Streak out *E. coli* cloning strain on LB agar and incubate at 37°C for 14-16 h.
2. Inoculate 10 mL of LB broth with a single colony and incubate at 37°C, 200 rpm for 14-16 h.
3. Transfer 0.5 mL of overnight culture to 50 mL of LB broth and incubate at 37°C, 200 rpm until OD_600 nm_ = 0.5-0.6.

TIP! While culturing, pre-chill 0.1 M CaCl_2_ and 0.1 M CaCl_2_, 15% (v/v) glycerol in an ice slurry bath.

1. Rapidly cool the culture by submersion of the flask in an ice slurry bath and incubate for 5-10 min with occasional mixing.

TIP! Keep cells on ice during the rest of procedure.

1. Harvest cells by centrifugation at 4,000 x *g*, 4°C for 5 min.
2. Discard supernatant and resuspend pellet in 5 mL of ice-cold filter-sterilised 0.1 M CaCl_2_.
3. Incubate suspension on ice for 15-20 min.
4. Harvest cells by centrifugation at 4,000 x *g*, 4°C for 5 min.
5. Discard supernatant and resuspend pellet in 2.5 mL of ice-cold filter-sterilised 0.1 M CaCl_2_, 15% (v/v) glycerol.
6. For each 100 µL of competent cells, add 10 µL of GG reaction and mix gently.
7. Aliquot suspension into 100 µL volumes in a sterile 96-well PCR plate kept in an ice slurry bath.
8. Seal with a sterile 96-well plate cover and transfer to -80°C freezer for > 30 min.

**Transformation of *E. coli***

1. Thaw 96-well PCR plate in an ice slurry bath for 10 min.
2. Incubate at 42°C for 45 s in a water bath.
3. Incubate in ice slurry bath for 2 min.
4. Transfer up to 1 mL of cells to 10 mL of LB broth in 50 mL centrifuge tube.

TIP! If you have more than 1 mL of cells, transfer to multiple LB tubes.

1. Recover cells at 37°C, 200 rpm for 1 h.
2. Harvest cells by centrifugation at 4,000 x *g*, room temperature for 5 min.
3. Resuspend in 1/10^th^ the volume of LB broth.

TIP! We find that we can plate up to 20,000 colonies on a single LB agar plate, and therefore for large libraries we recommend resuspending in 1/50^th^ volume of LB to reduce agar plate usage.

1. Plate all suspension on LB agar plates supplemented with 50 µg/mL apramycin (100 µL cells per plate).

TIP! Perform 10^-1^ and 10^-2^ dilutions of a small amount of the cell suspension in LB broth and plate 100 µL of each for colony counting.

1. Spread plates until dry and incubate at 37°C for 14-16 h.

**Isolating plasmid library**

1. Transfer 1 mL of LB broth to the surface of a transformant plate.
2. Resuspend colonies using a sterile plate spreader.
3. Transfer a further1 mL of LB broth to the surface of the agar and while holding the plate at an angle wash cells down to the bottom for collection.
4. Transfer suspension to 50 mL centrifuge tube.
5. Repeat this process with all transformant plates.

HINT! Pool suspensions into the same 50 mL centrifuge tube or sterile centrifuge bottle.

1. Measure OD_600 nm_ of the cell suspension.
2. Add 1/5^th^ volume of sterile glycerol and mix well.
3. Aliquot into cryovials and store samples at -80°C.
4. Inoculate 50 mL LB broth supplemented with 50 µg/mL apramycin with at least 4x10^8^ CFU of *E. coli* library.

HINT! For *E. coli,* 1 OD_600 nm_ ≈ 8x10^8^ CFU/mL

1. Incubate culture at 37°C, 200 rpm for 14-16 h.
2. Isolate plasmid using commercial miniprep kit.

**Mobilisation of library into ET12567/pUZ8002**

Using the above protocols, mobilise the library into conjugative *E. coli* strain ET12567/pUZ8002. Again, we aim for >50 colonies per clone. We find that the transformation of a 100 µL aliquot of ET12567/pUZ8002 with 500 ng of the plasmid library yields at least 5000 colonies. We always supplement media with 25 µg/mL chloramphenicol and 25 µg/mL kanamycin when working with ET12567/pUZ8002.

**Mass conjugation into *Streptomyces***

Aim for >50 transconjugants per sgRNA clone. We generally find that we recover >5,000 transconjugants per conjugation when using *Streptomyces albidoflavus* J1074 (10^9^-10^10^ spores/mL). For one conjugation:

1. Inoculate 33 mL of LB broth supplemented with 50 µg/mL apramycin, 25 µg/mL kanamycin, 25 µg/mL chloramphenicol using 4x10^8^ CFU of ET12567/pUZ8002 plasmid library.
2. Incubate culture at 37°C, 200 rpm until OD_600 nm_ = 0.5-0.6 (approximately 10-12 h).
3. Harvest cells by centrifugation at 4,000 x *g*, room temperature for 5 min.
4. Discard supernatant and resuspend pellet in 10 mL LB broth.
5. Harvest cells by centrifugation at 4,000 x *g*, room temperature for 5 min.
6. Repeat steps 4 and 5 twice.
7. Discard supernatant and resuspend pellet in 1 mL LB broth.
8. Add 100 µL *Streptomyces* spore stock and mix by aspiration.
9. Harvest cell mass by centrifugation at 4,000 x *g*, room temperature for 5 min.
10. Resuspend pellet in 300 µL of LB broth.
11. Plate suspension on Soya Flour Mannitol agar supplemented with 10 mM MgCl_2_ and 10 mM CaCl_2_.
12. Allow plate to dry in sterile cabinet and incubate at 30°C for 16-18 h.
13. Flood plate with 1 mL sterile water containing 2.5 µg/mL apramycin and 0.5 µg/mL nalidixic acid.
14. Allow plate to dry in sterile cabinet and incubate at 30°C until sporulation observed (typically 4-6 days for *S. albidoflavus* J1074).
15. Resuspend spores in 2-3 mL sterile 20% (v/v) glycerol using a sterile cotton bud.
16. Mix by aspiration and transfer to cryovials and store at -80°C.
17. Perform 10-fold dilution series of spore stocks in sterile PBS and plate 100 µL of each dilution on SFM supplemented with 25 µg/mL nalidixic acid and 50 µg/mL apramycin.
18. Incubate at 30°C until colonies observed.
19. Count colonies and calculate number of spores per mL.

**Analysing *Streptomyces* library**

1. Inoculate liquid broth supplemented with 25 µg/mL nalidixic acid and 50 µg/mL apramycin using 4x10^8^ library spores.
2. Incubate at 30°C, 200 rpm until cultures are turbid.
3. Harvest mycelia by centrifugation at 4,000 x *g*, room temperature for 5 min.
4. Resuspend pellet in 1 mL of 50 mM Tris pH 8 containing 100 µg/mL RNase A.
5. Aliquot into 400 µL volumes and add 40 µL of 100 mg/mL lysozyme.
6. Incubate at 37°C for 1-3 h until lysis is observed.
7. Add 50 µL of 10% (w/v) SDS and 150 µL 4 M NaCl and mix by aspiration.
8. Mix further by inversion and incubate on ice for >10 min.
9. Add 500 µL of phenol:chloroform:isoamyl alcohol and vortex for 10 s.
10. Separate phases by centrifugation at 16,000 x *g*, room temperature for 5 min.
11. Gently remove aqueous layer by aspiration.
12. Repeat steps 9-11 twice.
13. Transfer aqueous layer to a fresh microfuge tube.
14. Add 2.5x volumes of ethanol and shake gently to mix.
15. Pellet genomic DNA by centrifugation at 16,000 x *g*, room temperature for 10 min.
16. Gently remove supernatant by decanting.
17. Gently add 1 mL 70% (v/v) ethanol.
18. Centrifuge for at 16,000 x *g*, room temperature for 1 min.
19. Repeat steps 16-18 twice.
20. Decant supernatant and centrifuge at 16,000 x *g*, room temperature 1 min.
21. Gently remove residual ethanol by aspiration.
22. Leave to air dry in fume hood for >30 min.
23. Resuspend pellet in 100 µl of ultrapure water.
24. Quantify DNA via UV spectrophotometry.
25. Perform DNA digests as follows:

| Component | Stock concentration | Final amount | Volume |
| --- | --- | --- | --- |
| Genomic DNA  SbfI  Cutsmart buffer  Water | X ng/µL  20 U/µL  10x  - | 10 µg  10 U  1x  - | X µL  0.5 µL  5 µL  To 50 µL |

HINT! Setup enough reactions to digest 200-300 µg of genomic DNA.

1. Incubate reactions at 37°C for >12 h.
2. Add 1/5^th^ the volume of 6x DNA loading dye to the reactions.
3. Separate reactions by agarose gel electrophoresis.
4. Excise gel slice corresponding to DNA fragments between 3,500-5,000 bp.
5. Perform gel extraction using a Qiagen gel extraction kit.
6. Quantify DNA fragment via UV spectrophotometry.
7. Setup PCR reactions as follows:

| Component | Stock concentration | Final amount | Volume |
| --- | --- | --- | --- |
| Q5 reaction buffer  dNTPs  JEC134  JEC135  DNA fragment  Q5 DNA Polymerase  Water | 5x  10 mM each  10 µM  10 µM  X ng/µL  2 U/µL  - | 1x  0.5 mM each  0.5 µM  0.5 µM  500 ng  2 U  - | 20 µL  2 µL  5 µL  5 µL  X µL  1 µL  To 100 µL |

HINT! It is possible that you may not get enough purified DNA fragment for 500 ng. If this is the case, then use all the DNA you can. Ensure that for all samples tested in one experiment use the same amount of template.

1. Perform following reaction in thermal cycler:

| 98°C for 2 min |  |
| --- | --- |
| 98°C for 30 s |  |
| 71°C for 30 s | Repeat 24x |
| 72°C for 30s |  |
| 72°C for 2 min |  |
| 4°C hold |  |

1. Separate reactions via agarose gel electrophoresis.
2. Perform gel extraction using a Qiagen gel extraction kit.
3. Quantify DNA via UV spectrophotometry.
4. Sequence sample by NGS.
