# Supplementary material for: A platform for CRISPRi-seq in Streptomyces albidoflavus
Source: mBio. 2026 Jan 12;17(2):e03065-25. doi: 10.1128/mbio.03065-25 (PMC12892944; doi:10.1128/mbio.03065-25)
Supplement: Supplemental Figures — Figures S1–S5 and captions for supplemental tables. [file mbio.03065-25-s0002.docx]

**SUPPLEMENTARY INFORMATION**

**A platform for CRISPRi-seq in *Streptomyces* *albidoflavus***

Justin E. Clarke^1^, Tabitha R. Faulkner^1^, Ryan F. Seipke^1^*

^1^Astbury Centre for Structural Molecular Biology, Faculty of Biological Sciences, University of Leeds, Leeds, LS2 9JT, UK

**Supplementary Table 1:** TransportDB2.0-identified membrane transport genes harboured by *S. albidoflavus* J1074

**Supplementary Table 2:** Predicted transcription unit architecture for transporter genes in *S. albidoflavus* J1074

**Supplementary Table 3:** single-guide RNAs utilised during CRISPRi-seq

**Supplementary Table 4:** DESeq2 analysis of transporter CRISPRi-seq in *S. albidoflavus* J1074

**Supplementary Table 5:** Annotation of genes within positive-fitness TUs.

**Supplementary Table 6:** Bacterial strains and plasmids used in this study

**Supplementary Table 7:** Oligonucleotides used in this study

**Supplementary Figure 1:** Optimisation of induction conditions for the aTc-Theo switch in *Streptomyces albidoflavus* J1074

**Supplementary Figure 2:** Rarefaction curves of CRISPRi-seq libraries before and after mobilisation into S. albidoflavus J1074.

**Supplementary Figure 3:** Location of sgRNA binding within a gene does not affect CRISPRi efficiency in *Streptomyces albidoflavus* J1074

**Supplementary Figure 4:** Schematic representation of the F-type ATP synthase.

**Supplementary Figure 5:** Agar confirmation that that XNR_1463-to-XNR_1461 is positive to fitness.

**
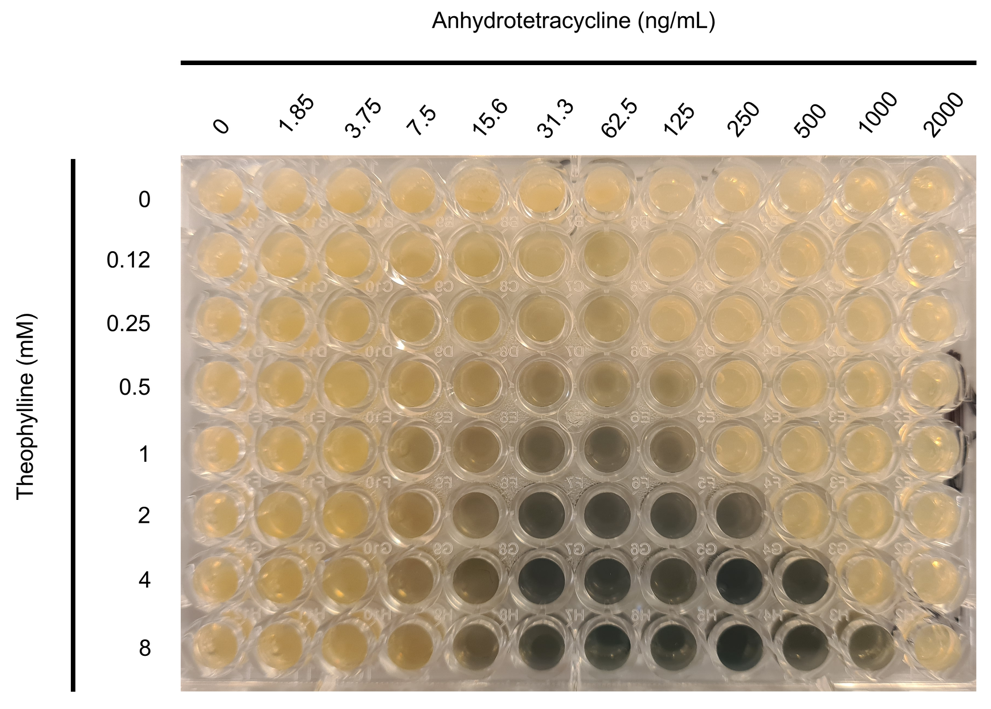
**

**Figure 1: Optimisation of induction conditions for the aTc-Theo dual switch in *Streptomyces albidoflavus* J1074.** Approximately 10^5^ spores of *S. albidoflavus* J1074 pIndigoC31Hyg-aTc-Theo were spotted on to ISP-2 agar in 96-well plates containing varying concentrations of anhydrotetracycline and theophylline, followed by incubation at 30°C for 3 d.


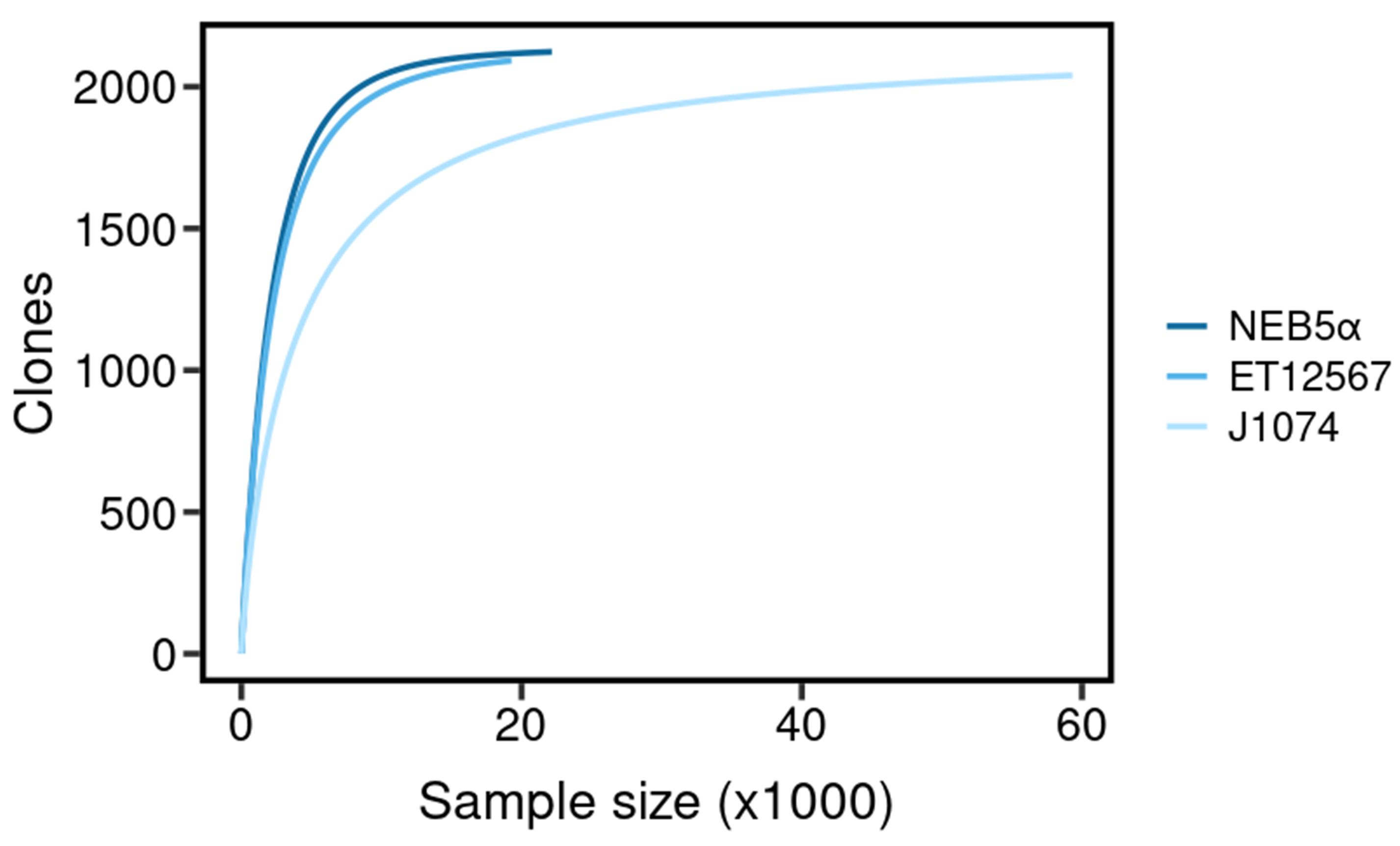


**Supplementary Figure 2:** Rarefaction curves of CRISPRi-seq libraries before and after mobilisation into *S. albidoflavus* J1074. Rarefaction curves were generated by Vegan 2.8.0 using the default parameters (45).


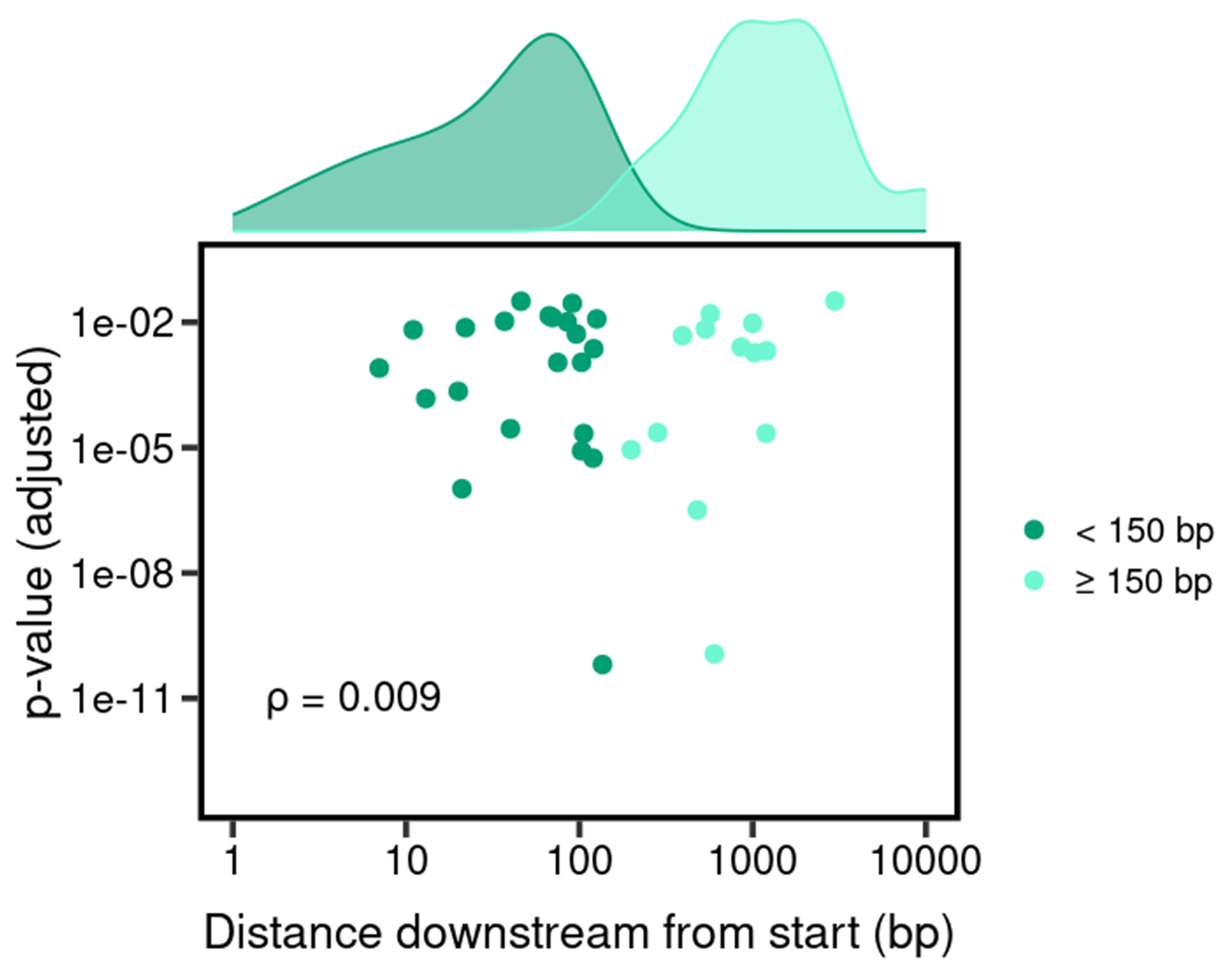


**Supplementary Figure 3: Location of sgRNA binding within a gene does not affect CRISPRi efficiency in J1074.** Spearman’s rank correlation coefficient between distance of sgRNA-binding site (relative to the putative start codon) and *P*-value (adjusted), for all sgRNA hits of positive-fitness genes. Density plots to show populations of sgRNA hits that bind < 150 bp and ≥ 150 bp relative to start of gene target.


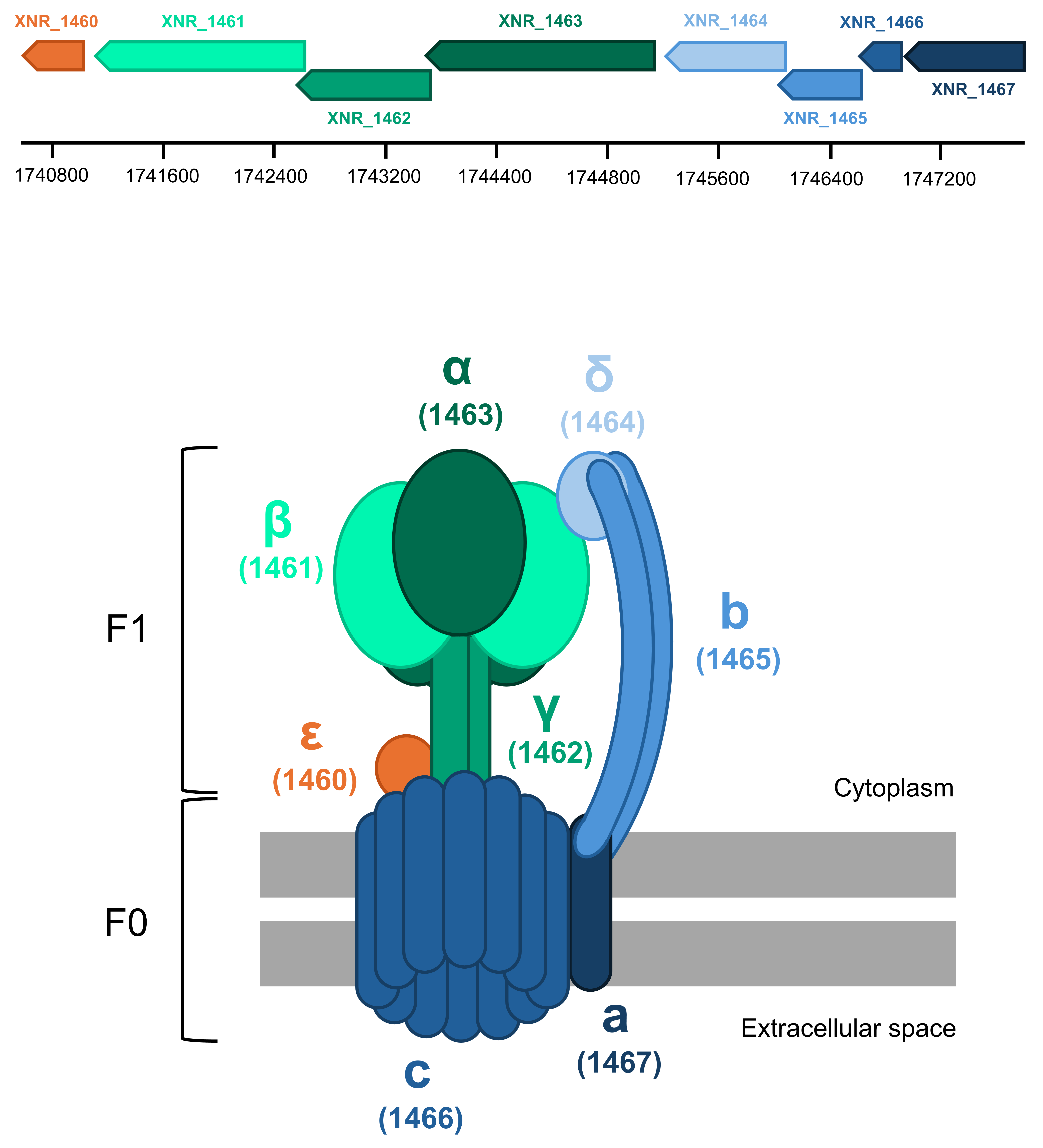


**Supplementary Figure 4:** Schematic representation of the F-type ATP synthase. Top, genetic loci and approximate coordinates. Bottom, cartoon of the F-type ATP synthase composed of XNR_1460, XNR_1461, XNR_1462, XNR_1463, XNR_1464, XNR_1465, XNR_1465, XNR_1466, XNR_1467.

**
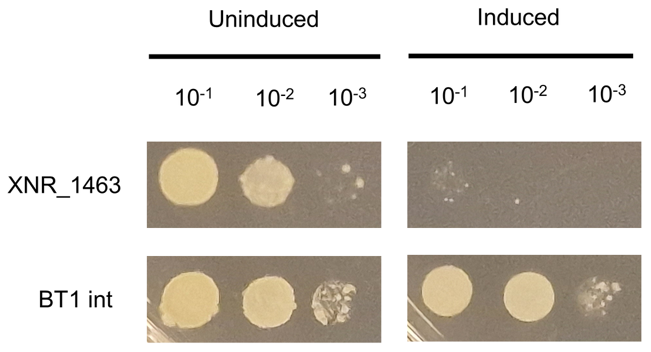
**

**Supplementary Figure 5: Agar confirmation that that XNR_1463 is positive to fitness.** Spores were as shown and spotted atop minimal media agar, followed by incubation at 30°C for 5 d. BT1 int, ΦBT1 integrase gene.

**Supplementary references**

45. Oksanen J, Simpson G, Blanchet F, Kindt R, Legendre P, Minchin P, O’Hara R, Solymos P, Stevens M, Szoecs E, *et al.* 2025. Vegan: Community Ecology Package. R package version 2.8.0. <https://vegandevs.github.io/vegan>

46. Chater KF, Wilde LC. 1976. Restriction of a bacteriophage of *Streptomyces* *albus* G Involving endonuclease SalI. J Bacteriol 128:644–650. <https://doi.org/10.1128/jb.128.2.644-650.1976>
